# Endophilin-A2-mediated endocytic pathway is critical for enterovirus 71 entry into caco-2 cells

**DOI:** 10.1080/22221751.2019.1618686

**Published:** 2019-05-28

**Authors:** Sheng-Lin Chen, Yan-Gang Liu, Yong-Tao Zhou, Ping Zhao, Hao Ren, Man Xiao, Yong-Zhe Zhu, Zhong-Tian Qi

**Affiliations:** a Department of Microbiology, Shanghai Key Laboratory of Medical Biodefense, Second Military Medical UniversityShanghai, People’s Republic of China; b General Hospital of the Tibet Military Area Command, Tibet, People’s Republic of China; c Company 7, Department of Clinical Medicine, Second Military Medical UniversityShanghai, People’s Republic of China

**Keywords:** Enterovirus 71, viral entry, endophilin, intestinal epithelial cells, endocytosis

## Abstract

Enterovirus 71 (EV71) is typically transmitted by the oral-faecal route and initiates infection upon crossing the intestinal mucosa. Our limited understanding of the mechanisms by which it crosses the intestinal mucosa has hampered the development of effective therapeutic options. Here, using an RNA interference screen combined with chemical inhibitors or the overexpression of dominant negative proteins, we found that EV71 entry into Caco-2 cells, a polarized human intestinal epithelial cell line, does not involve clathrin- and caveolae-dependent endocytic pathways or macropinocytosis but requires GTP-binding protein dynamin 2 and cytoskeleton remodelling. The use of siRNAs targeting endophilin family members revealed that endophlin-A2 is essential for the uptake of EV71 particles by Caco-2 cells. Subcellular analysis revealed that internalized EV71 virions largely colocalized with endophilin-A2 at cytomembrane ruffles and in the perinuclear area. Combined with viral entry kinetics, these data suggest that EV71 enters Caco-2 cells mainly via an endophilin-A2-mediated endocytic (EME) pathway. Finally, we showed that internalized EV71 virions were transported to endosomal sorting complex required for transport (ESCRT)-related multivesicular bodies (MVBs). These data provide attractive therapeutic targets to block EV71 infection.

## Introduction

Enterovirus 71 (EV71) is a non-enveloped, single-stranded, positive-sense RNA virus within the enterovirus genus of the *Picornaviridae* family. EV71 is one of the primary pathogenic agents that cause hand, foot, and mouth disease (HFMD) which has a wide spectrum of clinical manifestations, including persistent fever, herpangina, and lymphopenia [1]. Although symptoms in most patients are mild and self-limiting, severe neurological diseases, acute flaccid paralysis, and cardiopulmonary failure have been reported in some cases [2]. Despite several vaccine candidates whose widespread utilization is limited due to their limitations in cross-protection, there are currently no effective prophylactic or therapeutic agents for EV71 infection [3–5] As EV71 is typically transmitted by the oral-faecal route and initiates infection upon crossing the intestinal mucosa, a better understanding of the cellular factors that influence virus invasion of enterocytes would aid in the development of new therapeutics options.

EV71 shows distinct internalization routes in different host cells, partly because of the diversity of the EV71 surface receptor. Several studies have suggested that EV71 enter rhabdomyosarcoma (RD) cells and NIH3T3 cells through a clathrin-dependent pathway, while it enters Jurkat and L-PSGL-1 cells in a caveolae-mediated pathway [6]. Moreover, a recent study using endocytosis inhibitors found that disrupting clathrin and dynamin did not inhibit, but rather promoted, EV71 infection in A549 cells, suggesting an undefined dynamin-independent endocytic pathway that mediates the infectious entry of EV71 [7]. By spreading through the oral-faecal route, EV71 initiates its replication cycle in human intestinal cells. However, the precise mechanism of the endocytosis necessary for EV71 entry into intestinal cells remains unknown.

In host cells, cargo, such as receptors at the plasma membrane, can be internalized and delivered to multivesicular bodies (MVBs), a cell compartment containing luminal vesicles that invaginate and bud from the limiting membranes of late endosomes [8]. The emergence of MVBs critically depends on the ordinal assembly of the endosomal sorting complex required for transport (ESCRT)-0, -I, -II, -III complexes and the catalyzing disassembly of the ESCRT-III complex by AAA ATPase VPS4A and B [9]. The potential roles of ESCRT-MVBs in the viral life cycle, including viral entry, transport, and budding, has been widely investigated. For example, several viruses, including human immunodeficiency virus (HIV), Crimean-Congo haemorrhagic fever virus (CCHFV), Lassa fever virus (LASV), vesicular stomatitis virus (VSV), and influenza A virus have been proven to traffic through MVBs and their ESCRT sorting machinery during the early stages of infection [10–13] A recent study also discovered that hepatocyte growth factor-regulated tyrosine kinase substrate (HRS), a key component of ESCRT-0, is required for endosomal sorting of membrane proteins into MVBs and is also essential for TLR7 signalling to orchestrate immunity and inflammation during EV71 infection [14]. However, it remains unclear if ESCRT-MVBs play a role in EV71 entry and transport.

Here, using a single round, robust high-throughput siRNA screen and subsequent validation and exploration methods, we investigated the internalization mechanism of EV71 into Caco-2 cells, a polarized human epithelial colorectal adenocarcinoma cell line that serves as an in vitro model of the intestinal epithelium. Unlike the viral entry mechanisms described to date, EV71 utilizes the endophilin-A2-mediated endocytic (EME) pathway as its major endocytic infection route in intestinal epithelial cells and can be transported through ESCRT-MVBs.

## Materials and methods

### Cells and virus

Caco-2 (ATCC HTB-37, Manassas, USA), RD (ATCC CCL-136), 293T/17 (ATCC CRL-11268), SH-SY5Y (ATCC CRL-2266), A549 (ATCC CCL-185) and Huh7 (Cell Biology, Chinese Academy of Sciences, Shanghai, China) were maintained in a Dulbecco’s Modified Eagle Medium (DMEM) (Thermo Fisher scientific, NY, USA) supplemented with 10% foetal bovine serum (FBS) (GIBCO, NY, USA) at 37°C in a 5% CO_2_-humidified environment. The Jurkat T cell line (ATCC TIB-202) was maintained in RPMI-1640 Medium (ATCC) containing 10% FBS (GIBCO Invitrogen). The EV71 strain (FJ08089) was isolated from an 8-year old boy with a confirmed case of HFMD in Fujian province, China and was propagated in RD cells. Viral titres were determined by TCID_50_ in RD cells.

### Antibodies, chemical inhibitors, small interfering RNAs (siRNAs), and plasmids

Mouse anti-enterovirus 71 VP1 monoclonal antibody (ab36367), rabbit anti-clathrin heavy chain (ab21679), anti-dynamin 2 (ab3457), and anti-caveolin-1(ab2910) polyclonal antibodies were purchased from Abcam (Cambridge, England). Rabbit anti-GAPDH monoclonal antibody (2118S) was purchased from Cell Signaling Technology (Massachusetts, USA). Mouse anti-endophilin-A2 monoclonal antibody was purchased from Santa Cruz (sc-365704, Texas, USA). Rabbit anti-endophilin-A2 polyclonal antibodies (GTX113548) and rabbit anti-enterovirus 71 VP1 polyclonal antibody (GTX132339) were purchased from GeneTex (Texas, USA). Rabbit polyclonal antibody against SCARB2 (NB400-102) was purchased from Novus Biologicals (Colorado, USA). Mouse anti-human CD162 (PSGL-1) antibody (556053) was purchased from BD Biosciences (California, USA). Alex Fluor 488 (AF-488)-conjugated donkey anti-mouse IgG, HRP-conjugated goat anti-rabbit IgG, and HRP-conjugated goat anti-mouse IgG were purchased from Thermo Fisher scientific.

Chemical inhibitors used in the drug inhibition assays include chlorpromazine (CPZ) (Sigma-Aldrich, Missouri, USA), Dynasore (Selleck, Texas, USA), IPA-3 (Selleck), filipin III (Santa Cruze Biotechnology), bisindolylmaleimide (Cell Signaling Technology), NSC-23766 (Sigma-Aldrich), cytochalasin D (Sigma-Aldrich), jasplakinolide (Sigma-Aldrich), and latrunculin A (Sigma-Aldrich). The specificities of the inhibitors are listed in Table S2.

Specific siRNAs targeting VPS37A (5’-CCAGAUGUCCCUGAUGCAUTT-3’), VPS37C (5’-GGACCUUGUUAGACCUUCUTT-3’), and CHMP2A (5’-GCGCUAUGUGCGCAAGUUUTT-3’) were synthesized by GenePharma (Shanghai, China). Human Membrane Trafficking siRNA library (G-005505, 140 targets) and siEndophilin-A1 (HSS109708), -A2 (HSS109705), -A3 (HSS109711), -B1 (D-015810-02-0002), and -B2 (L-017086-00-0005) were purchased from Dharmacon-Thermo Fisher Scientific.

Plasmid constructs expressing GFP tagged wild-type (WT) and K44A dominant negative (DN) dynamin II were kindly provided by Mark McNiven (Mayo Institute, Rochester, MN). The EPS 15 WT and DN (△95/295) constructs were kindly provided by A. Benmerah (INSERM, Paris, France). The GFP-tagged constructs expressing WT and DN mutants caveolin-1 were kindly provided by J. M. Bergelson (University of Pennsylvania) [15].

### Western blotting

Cells were quickly collected in radioimmunoprecipitation assay (RIPA) buffer (Thermo Fisher Scientific), sonicated, and boiled. The samples were normalized to equal concentrations of protein, run on 10% SDS-PAGE, transferred to polyvinylidene difluoride (PVDF) membranes (Bio-Rad, California, USA), blocked in 5% skimmed milk, incubated with the indicated primary antibodies and secondary-HRP conjugated antibodies. The membranes were visualized with the ECL Plus enhanced chemiluminescence western blotting detection reagents (PerkinElmer Life Sciences, Massachusetts, USA) depending on the strength of the signals.

### Immunofluorescence

Cell immunostaining was performed as previously described [16]. Briefly, Caco-2 cells plated on chambered coverglass (Thermo Fisher Scientific) were fixed in 4% PFA in PBS for 15 min at room temperature, permeabilized with 0.1% Triton X-100, and blocked for 2 h with 3% BSA in PBS. For experiments using EIPA, cells were fixed in cold methanol for 20 min at −20°C. After incubation with primary antibodies (overnight at 4°C) and fluorescent secondary antibodies or tetramethylrhodamine-conjugated Phalloidin (ATT Bioquest, California, USA) (1 h at room temperature), nuclei were counterstained with 4’, 6’-diamidino-2-phenylindole (DAPI, Roche, Basel, Switzerland). Chambers were observed with an Olympus IX 81 fluorescence microscope, and for cellular localization analysis, with a Zeiss or Leica SP8 confocal fluorescence microscope. Images were adjusted for brightness and contrast, and figures were assembled with Photoshop CS4.

### Quantitative real time PCR (qRT-PCR)

Total RNA was extracted using TRIzol (TaKaRa, Shiga, Japan) according to the manufacturer’s manual. cDNA was synthesized using PremeScriptTM RT Master Mix (TaKaRa) according to the protocol recommended by the manufacturer. The synthesized cDNA was subjected to quantitative PCR analysis with SYBR Premix Ex Taq (TaKaRa) using respective primer pairs (Table S3) for each gene. Genome copy numbers were normalized to GAPDH levels by using the comparative cycle threshold values determined in parallel. Data were analyzed relative to controls. All assays were performed on an ABI 7300 system (Applied Biosystems, Massachusetts, USA).

### EV71 single round virus infection

All of the plasmids used for EV71 pseudotyped virus [EV71(FY)-Luc] generation were kindly provided by Wenhui Li (National Institute of Biological Sciences, China). EV71 (FY)-Luc was generated as previously described [17], and the titre was determined in RD cells. For EV71 single round viral infection assays, 20 μl of EV71 (FY)-Luc (∼10^7^ copies of viral genome) was mixed with 80 μl of fresh complete medium supplemented with 10% FBS, then added to cells on a black clear 96-well plate. After incubation for 2 h at 37°C, cells were washed once with PBS and fresh medium was added. Firefly luciferase activity was measured following the Luciferase Assay System manual (Promega, Wisconsin, USA) at 24 h p.i. For measurement of luciferase activity in Jurkat T cells, 100 μl of EV71 (FY)-Luc was added to cells cultured in flasks (1 × 10^5^ cells/ml, 2.5 ml of total volume). After incubation for 24 h at 37°C, cells were collected in tubes and centrifuged at 1000 rpm for 10 min. Cells were then washed twice with PBS and centrifuged again. Firefly luciferase activity was measured for every 5 × 10^4^ Jurkat T cells following the Luciferase Assay System manual.

### Drug inhibition assays

For drug inhibition experiments, Caco-2 cells were seeded in collagen-coated 96-well plates (5 × 10^4^ cells/well) and pre-treated with chemical inhibitors at different concentrations (Table S2) for 60 min at 37°C. Following this treatment, cells were incubated with EV71 (MOI = 10) or EV71 (FY)-luc (∼10^7^ copies of viral genome) for 2 h at 37°C in a concentration of 10% BD NuSerumTM medium containing specific inhibitor. The EV71-infected cells were visualized by immunofluorescence at 48 h p.i. The firefly luciferase activity was measured at 24 h p.i. with EV71 (FY)-luc.

For the time-of-addition studies with chemical inhibitors, cells were seeded in collagen-coated 96-well plates (5 × 10^4^ cells/well) and incubated with EV71 (MOI = 10) at 4°C for 1 h to allow virus binding then shifted to 37°C. At different times before or after virus binding, cells were treated with Dynasore (80 μM). After incubation for 1 h, the drug solution in each well was replaced by fresh culture medium. At 48 h p.i, the EV71-infected cells were visualized by immunofluorescence.

### Transferrin, CT-B uptake, and dextran uptake assay

AF-488 transferrin (C34776, Molecular Probes, Minnesota, USA), AF-555 transferrin (T35352, Molecular Probes), or AF-555 CT-B (T13342, Molecular Probes) was added to cells for 20 min at 37°C and removed from the cell surface by washing with 0.1 M glycine, 150 mM NaCl (pH 2.5) prior to fixation. AF-555 Dextran (D34679, Molecular Probes) was diluted in PBS to 1 mg/ml and added to the cells for 30 min. After washing three times with PBS, the samples were fixed with methanol at −20°C for 20 min, washed with PBS, and viewed by fluorescence microscopy.

### Cell viability assay

Cell viability was evaluated using a CCK-8 cell counting assay kit (Dojindo, Kumamoto, Japan.) following the manufacturer’s instructions. Cells were seeded at a density of 5 × 10^4^ cells/well in a 96-well plate and allowed to adhere overnight. Following treatment with drugs for 2 h or siRNAs for 60 h, the cells were washed twice with PBS. CCK-8 solution diluted in DMEM (no phenol red) was then added to each well, and the plates were incubated for an additional 2 h at 37°C. Formation of the coloured formazan product was assessed by monitoring the absorption at 450 nm using a microplate reader (BioTek, Vermont, USA). The percentage of viable cells was calculated using the formula: ratio (%) = [OD (Treatment)–OD (Blank)] / [OD (Control)–OD (Blank)] × 100. The control group was cell culture medium containing the same concentration of drugs or non-targeting siRNA. Each sample was assayed with six replicates per assay, and each experiment was repeated three times.

### Viral kinetics assay

Viral kinetics assays were conducted as described previously [18]. Briefly, to determine the rate of viral entry, Caco-2 cells were seeded 24 h prior to experimentation in collagen-coated 24-well plates (2 × 10^5^ cells/well), pre-treated with EV71 (MOI = 100) in 500 μl DMEM at 4°C for 1 h. The cells were washed with PBS, fresh culture medium was added, and the plates were transferred to 37°C. At the indicated times, cells were washed with PBS and treated with proteinase *K* (1 mg/ml) (Thermo Fisher Scientific) for 30 min at 4°C to remove adsorbed but not internalized virus. Proteinase *K* was inactivated with 2 mM phenylmethylsulfonyl fluoride (PMSF) in PBS with 3% bovine serum albumin (BSA). The cells were washed three times with PBS, and collected in 500 μl of TRIzol reagent per well for RNA isolation and qRT-PCR analysis. As a control, a parallel set of cultures was processed under the same conditions, except that proteinase K was replaced by PBS. For each time point, the mRNA level of virus VP1 expression in the PBS control group were set to 100%.

### Cell transfection and transient expression

For plasmid overexpression, cells were transfected according to the manufacturer’s instructions for Lipofectamine 2000 (Thermo Fisher Scientific). Brieﬂy, cells were seeded onto 24-well tissue culture plates and grown overnight until 75% confluent. Next, 0.8 μg of the plasmid construct was mixed with 50 μl of Opti-MEM (Thermo Fisher Scientific) for 5 min at room temperature. The mixture was then added to 50 μl of Opti-MEM containing 2 μl of Lipofectamine 2000 that had undergone similar incubation conditions. After a further incubation period of 20 min, the DNA-liposome complexes were added to the cells, which had been starved in Opti-MEM for 4 h before transfection. After incubation for 6 h at 37°C, 1 ml of maintenance medium was added, and the mixture was incubated for an additional 48 h before virus infection.

### RNA interference screen

The endocytic and membrane-trafficking genes were targeted by gene-specific siRNAs from the Human Membrane Trafficking siRNA Library (Dharmacon -Thermo Fisher Scientific; G-005505, 140 targets). The base of this library is a commercially available set of 140 siRNAs targeting genes known to be directly or indirectly involved in regulating different endocytic pathways, cytoskeleton rearrangement, phosphatidylinositol signalling, and vesicle/cargo trafficking. A smart pool approach of incorporating four siRNAs targeting each gene was used. The untreated cells, transfection reagent (Dharmacon FECT) alone, a non-targeting siRNA (Dharmacon -Thermo Fisher Scientific), and an siRNA on-target plus human SCARB2 (Dharmacon -Thermo Fisher Scientific) were used as controls. The RNAi screen was performed according to the manufacturer’s instructions. Briefly, Caco-2 cells were seeded in collagen-coated 96-well black optical-bottom plates (Nunc) (5 × 10^4^ cells/well). The following day, cells were transfected with siRNAs (75 nM per well) and incubated at 37°C for 60 h to ensure effective gene knockdown. Cells were washed once with PBS, infected with EV71 (MOI = 10) for 120 min, and maintained for an additional 48 h at 37°C. After infection, the cells were fixed and then subjected to immunofluorescence staining. Collation of image data was performed by the ArrayScan VTI HCS automated fluorescence microscope reader system (Cellomics). Data analysis after image acquisition was carried out using Cellomics Target Activation Bioapplication (Cellomics). DAPI-stained nuclei were counted to determine total cell populations, while AF-488-stained cytoplasm was counted to determine the number of virus-infected cells. To ensure that the screen had minimal signal variation, the *Z*’ score was determined as described previously [19]. The effects of siRNA knockdown of different endocytic genes were determined by the decrease in the percentage of viral antigen positive cells after EV71 infection. We utilized a 50% or greater reduction in the number of fluorescently stained EV71-infected cells in duplicate screens as an siRNA-induced effect that suppressed EV71 infection. Three independent screening assays were performed.

### Electron microscopy

Cryosections of cells were prepared as previously described [20]. Briefly, RD cells and Caco-2 cells were incubated with EV71 (MOI = 100) for 90 min and fixed by 4% PFA in 0.1 M NaPi buffer (pH 7.4) for 100 min. Cells were then rinsed in 20 mM glycine in PBS, embedded in 12% gelatine, infiltrated with 2.3 M sucrose, and frozen in liquid nitrogen. Ultrathin (50–60 nm) cryosections were cut on an EM UC7 ultramicrotome (Leica, Heerbrugg, Switzerland).

For immunolabelling, ultrathin cryosections on formvarcoated grids were quenched in 50 mM glycine/50 mM NH4Cl and labelled with rabbit anti-enterovirus 71 VP1 polyclonal antibody (GTX132339) and protein-A gold (PAG, 15 nm, Bioworld). Sections were examined with a transmission electron microscope HT7700 (Hitachi, Tokyo, Japan). Images were adjusted for brightness and contrast and figures were assembled with Photoshop CS4.

### Statistical analysis

All data in graphs are expressed as the arithmetic mean ± SEM of three independent experiments, and the statistical significance of differences were evaluated by one-way ANOVA followed by Bonferroni *post hoc* analysis using Excel (Microsoft) and GraphPad Prism 7.00 (GraphPad Software, California, USA). *P* < 0.05 was considered to be statistically significant.

## Results

### Identification of membrane-trafficking factors involved in EV71 infection using an siRNA screen

To identify cellular factors involved in EV71 infection in Caco-2 cells, we used a single round, robust high-throughput siRNA screening assay. Caco-2 cells were transfected with an siRNA library targeting 140 human membrane trafficking genes, followed by infection with EV71 60 h post-transfection (p.t). Immunofluorescence was then used to detect EV71 capsid VP1 antigen 48 h post-infection (p.i.). SiRNA targeting SCARB2 (human scavenger receptor class B, member 2), a functional receptor of EV71 [21], was included as a positive control. The verification of the function of SCARB2 in Caco-2 cells is shown in Fig S1. To detect the percentage of infected cells, automated microscopy and image analysis were utilized to calculate the number of infected cells and the total number of cells. A 50% or greater reduction in the number of infected cells compared to non-targeting siRNA was regarded as an siRNA-induced effect that inhibited EV71 infection.

**Figure 1.**
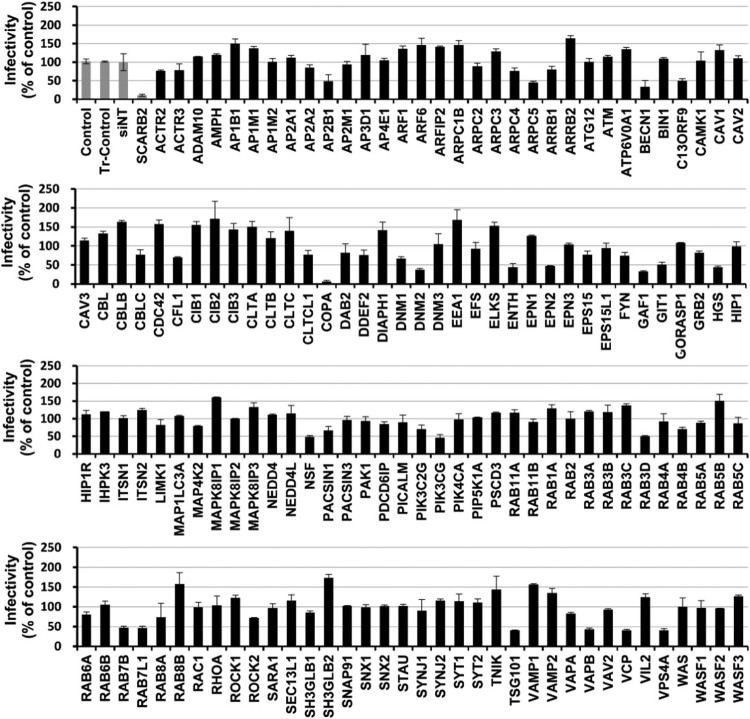
Identification of membrane-trafficking factors involved in EV71 infection Caco-2 cells were transfected with Human Membrane Trafficking siRNA library. Sixty hours post-transfection, the cells were infected with EV71 (MOI = 10) for 48 h before immunofluorescence assays were performed to detect EV71. The number of infected cells and the total cells were assessed by automated microscopy and image analysis. EV71-infected cells after siRNA knockdown were compared to controls and are presented as the percentage of viral antigen-positive cells. The transfection controls (Tr-control) were established as the baseline for infection and transfection efficiency. Genes showing a >50% decrease in EV71 infectivity compared to the control group were considered for further analysis. Values are normalized to control group. SiNT: non-targeting siRNA.

Among the 140 siRNAs, 20 were identified to suppress EV71 infection, as shown in Figure 1. A brief description of the reported functional roles for each of these genes is provided in Table S1. Cell viability was measured, and minimal cytotoxicity was observed (data not shown). The genes scored as positive were classified into several protein classes. Silencing of DNM2, a large GTPase which acts by mediating the release of newly formed endocytic vesicles from the plasma membrane, displayed an inhibitory effect on EV71 infection. Knockdown of genes encoding for ESCRT machinery [HGS, TSG101, VPS4A, and C13ORF9 (VPS36)] led to a decrease in EV71 infection. Depletion of kinase PIK3CG, which mainly mediates cellular signalling transduction, exhibited an inhibitory effect on virus infection. Knockdown of BECN1, involved in the regulation of autophagy, significantly decreased EV71 infection. Silencing of ARPC5, involved in actin polymerization, resulted in a reduction in virus infection. In addition, siRNAs targeting clathrin-mediated pathway-related genes, such as ENTH, EPN2, and AP2B1, led to a decrease in EV71 infection. Moreover, silencing of many genes involved in vesicle and endosomal transport, such as COPA, GAF1, GIT1, VAPB, VCP, NSF, RAB3D, RAB7B, and RAB7L1, also led to a decrease in viral entry.

### EV71 infection of Caco-2 cells is independent of clathrin, caveolin, and macropinocytosis

**Figure 2.**
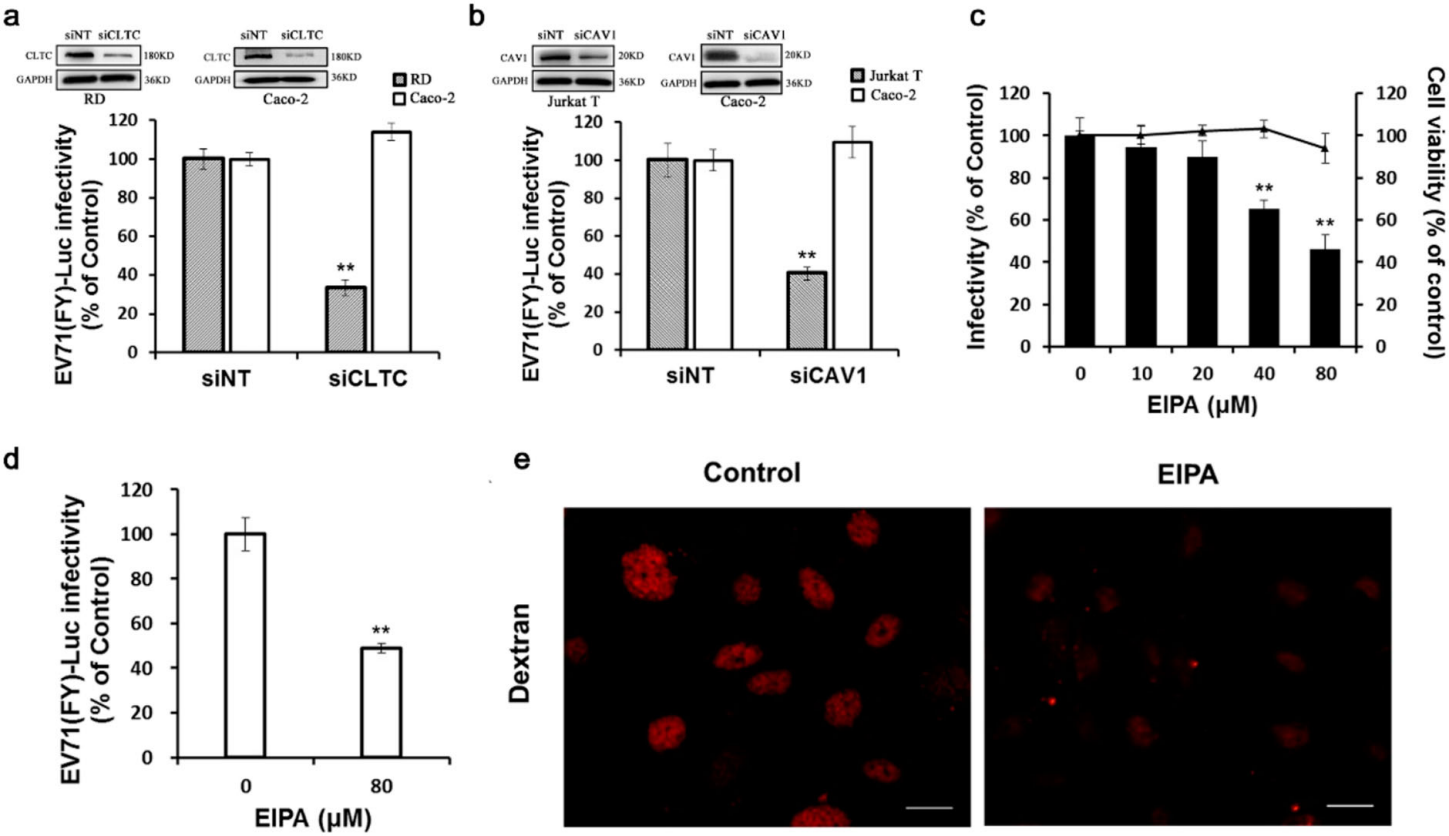
Clathrin, caveolin, and macropinocytosis are not involved in EV71 infection of Caco-2 cells (a, b) Cells transfected with siCLTC (a) or siCAV1 (b) were infected with EV71 (FY)-Luc. Protein levels of CLTC and CAV1 were measured by western blotting and EV71 (FY)-Luc infectivity was measured by firefly luciferase activity. Values are normalized to non-targeting siRNA (siNT) control. (c, d) The cells were pre-treated with EIPA at different concentrations then infected with EV71 (MOI = 10) or EV71 (FY)-Luc. EV71 infectivity was measured by immunofluorescence (c) and EV71 (FY)-Luc internalization was analyzed by firefly luciferase activity (d). Values are normalized to DMSO control. (e) Caco-2 cells were left untreated (control) or were treated with EIPA (100 μM) then incubated with 200 μg/ml AF 555-conjugated dextran (red) for 20 min at 37°C and examined by immunofluorescence microscopy. Scale bars, 100 μm. **p < 0.01.

Our RNAi screen indicated that, although silencing several factors (ENTH, EPN2 and AP2B1) involved in clathrin-mediated pathways resulted in a reduction in EV71 infectivity, siRNAs targeting CLTA, CLTB, CLTC, and AP1A1, four key factors mediating clathrin-dependent endocytosis, increased viral infectivity. To determine the role of clathrin in EV71 entry, pseudotyped virus particles EV71(FY)-Luc were used. As shown in Figure 2(a), depletion of CLTC with siRNA (siCLTC) did not affect EV71(FY)-Luc infectivity in Caco-2 cells. As a control, siCLTC inhibited EV71 entry into RD cells, whose mechanism of internalizing EV71 was previously reported to be through a clathrin-dependent endocytic pathway [22]. Thus, we presumed that clathrin-mediated internalization is not required for EV71 infection in Caco-2 cells. To further investigate the role of clathrin in EV71 entry into Caco-2 cells, specific inhibitor or DN constructs were used. Pre-treatment of Caco-2 cells with chlorpromazine (CPZ), which can specifically inhibit clathrin recycling and formation of clathrin coated-vesicles, did not exhibit an inhibitory effect on EV71 infection (Fig S2a). Expression of an EPS15 DN also did not result in a reduction of EV71 infectivity (Fig S2b). As a control, Caco-2 cells treated with CPZ or transfected with EPS15 DN showed a decreased uptake of AF 488-conjugated transferrin, an indicator of clathrin-mediated endocytosis (Fig S2c). Hence, a clathrin-dependent pathway is not required for EV71 infection.

We next investigated whether caveolae-mediated endocytosis is involved in EV71 infection. As shown in Figure 2(b), silencing of CAV1 decreased EV71 entry into Jurkat T cells but not into Caco-2 cells. Pre-treatment with filipin, which can inhibit caveolin-mediated endocytosis, did not inhibit EV71 infection (Fig S2d). In addition, the expression of caveolin-1 DN (CAV1 DN) also did not result in a reduction in EV71 infection (Fig S2e). As a control, cholera toxin B subunit (CT-B) internalization, usually used as a marker of caveolae-dependent endocytosis, was inhibited by filipin and caveolin-1 DN in Caco-2 cells (Fig S2f). Thus, these data suggest that EV71 infection in Caco-2 cells is caveolae-independent.

Macropinocytosis requires a variety of cellular factors, such as the sodium/hydrogen exchanger, Rho GTPase (CDC42 and RAC1), PKC, and p21-activated kinase 1 (PAK1). Since EIPA is known as an inhibitor of the sodium/hydrogen exchanger and can prevent membrane ruffling during macropinocytosis, we first determined whether EIPA could inhibit EV71 infection and entry. As shown in Figure 2(c), EIPA pretreatment significantly decreased viral infectivity in a dose-dependent manner and simultaneously caused a 51% reduction in EV71(FY)-Luc entry (Figure 2(d)). As a positive control, dextran uptake was also inhibited by EIPA (Figure 2(e)). However, our RNAi screen showed that silencing CDC42, RAC1, PKC, and PAK1 displayed no effect on EV71 infection in Caco-2 cells (Figure 1). To further determine the roles of these factors in EV71 infection, specific chemical inhibitors were used. Pre-treatment with NSC23766 (a RAC1 inhibitor) (Fig S3a), IPA-3 (a PAK1 inhibitor) (Fig S3b) and bisindolylmaleimide (a PKC inhibitor) (Fig S3c) did not decrease EV71 infectivity. Cell viability was not affected by all above inhibitors at all concentrations used. Taken together, these data indicate that while EV71 infection is EIPA-sensitive, it does not occur via micropinocytosis.

### Dynamin 2 and the actin cytoskeleton are necessary for EV71 entry

**Figure 3.**
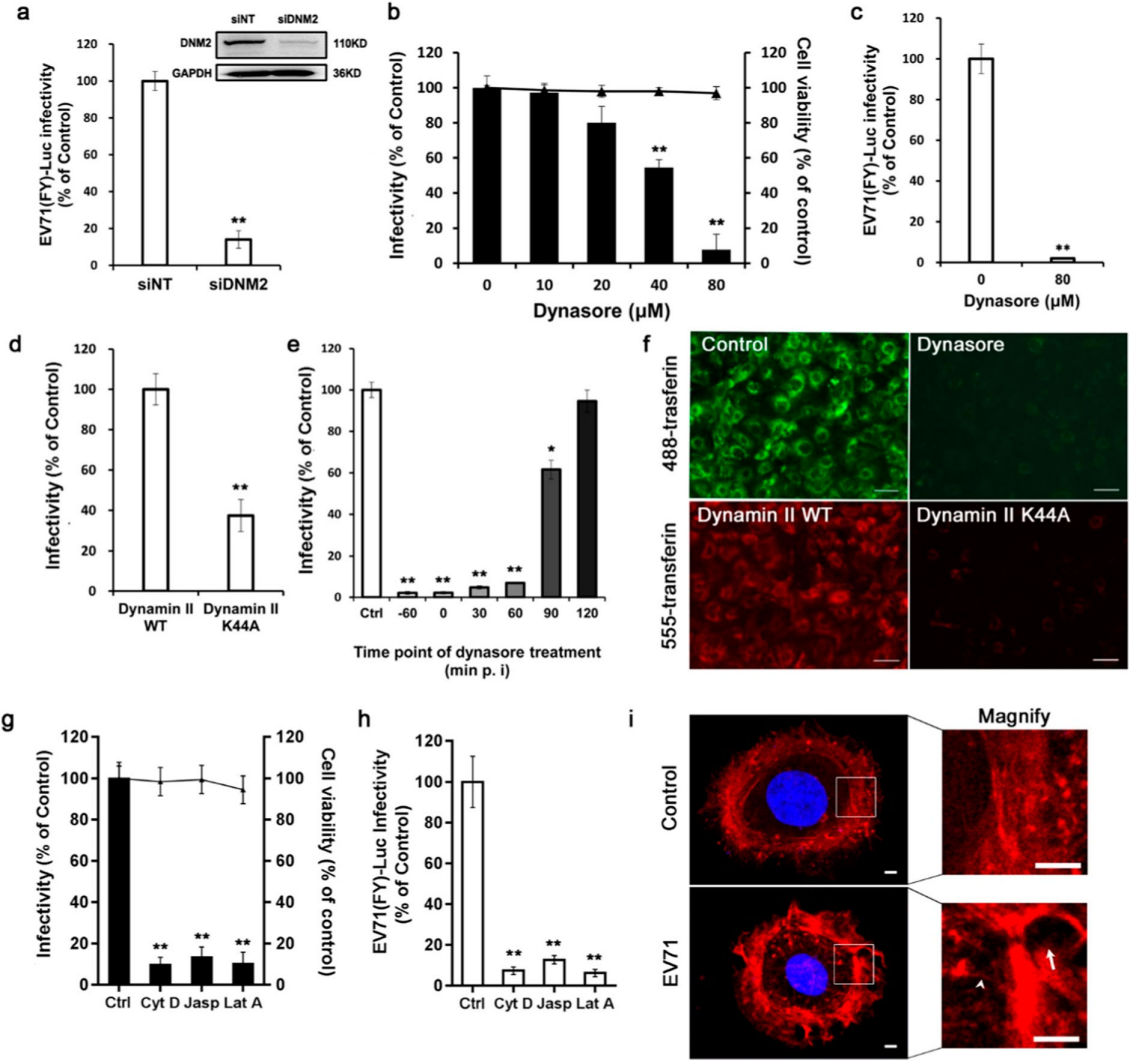
Dynamin 2 and the actin cytoskeleton are essential for EV71 entry (a) Caco-2 cells were transfected with siDNM2 then infected with EV71 (FY)-Luc. EV71 (FY)-Luc infectivity was measured by firefly luciferase activity. Silencing efficiency of siDNM2 was detected by western blotting. Values are normalized to non-targeting siRNA (siNT) control. (b, c) The cells were pre-treated with Dynasore at different concentrations then infected with EV71 (MOI = 10) or EV71 (FY)-Luc. EV71 infection was assessed by immunofluorescence, cell viability was tested by CCK-8 kit (b), and EV71 (FY)-Luc internalization was measured by firefly luciferase activity (c). Values are normalized to DMSO control. (d) Cells expressing wild-type (WT) or dominant-negative (DN) forms of dynamin 2 were infected with EV71 (MOI = 10) and infectivity was analyzed by immunofluorescence. Values are normalized to WT control. (e) Caco-2 cells bound with EV71 (MOI = 10) at 4°C for 1 h were transferred to 37°C. At the indicated times, cells were treated with Dynasore (80 μM). Forty-eight hours after transfer to 37°C, EV71 internalization was analyzed by immunofluorescence. −60, add Dynasore at the time of adding EV71. Values are normalized to DMSO control (Ctrl). (f) AF-488 transferrin or AF-555 transferrin was added to cells pre-treated with Dynasore or cells expressing DN/WT forms of dynamin 2 and examined by immunofluorescence microscopy. Scale bar, 150 μm. (g, h) The cells were pre-treated with cytochalasin D (Cyt D, 10 μM), latrunculin A (LatA, 5 μM), or jasplakinolide (Jasp, 5 μM) then infected with EV71 (MOI = 10) or EV71 (FY)-Luc. EV71 infectivity was assessed by immunofluorescence (g) and EV71 (FY)-Luc internalization was measured by firefly luciferase activity (h). Values are normalized to DMSO control (Ctrl). (i) Caco-2 cells exposed to EV71 (MOI = 100) or mock-treated for 90 min were stained with tetramethylrhodamine-conjugated phalloidin (red) and observed by confocal microscopy. White arrows indicate dorsal ruffles and arrowheads indicate microfilaments perpendicular to the surface of the cell. Nuclei were stained with DAPI. Insets show higher magnification of boxed areas. Scale bars, 20 μm. *p < 0.05, **p < 0.01.

Our siRNA screen showed that transfection of dynamin 2 siRNA into Caco-2 cells resulted in an approximately 89% reduction in EV71 infectivity. To confirm the role of dynamin 2 in EV71 entry, EV71(FY)-Luc was used. As shown in Figure 3(a), transfecting cells with dynamin 2 siRNA also markedly decreased firefly luciferase activity of EV71(FY)-Luc compared to the siNT control. The silencing efficiency of dynamin 2 siRNA was detected by western blotting. To further confirm this phenomenon, Caco-2 cells were pre-treated with Dynasore, a GTPase pharmacological inhibitor that can rapidly and reversibly inhibit dynamin. As shown in Figure 3(b) and (c), EV71 infection was inhibited by Dynasore in a dose-dependent manner, and EV71(FY)-Luc entry was almost completely blocked by 80 μM Dynasore. Furthermore, the expression of dynamin 2 DN exhibited a significant inhibitory effect on EV71 infection (Figure 3(d)). The functions of Dynasore and dynamin 2 DN were verified by their abilities to inhibit transferrin uptake (Figure 3(f)). To specifically investigate at which stage of EV71 entry dynamin 2 engages, Caco-2 cells were incubated with Dynasore (80 μM) prior to the start of infection or at the indicated times after infection with EV71. As shown in Figure 3(e), EV71 infection was significantly inhibited when Dynasore was added −60, 0, 30, and 60 min p.i., whereas the addition of Dynasore at 90 min p.i. or at later time points exhibited the least inhibitory effects on virus infection.

The actin cytoskeleton plays an essential role in several endocytic processes such as creating protrusions, supporting invagination of membrane segments, and triggering vesicle scission [23]. In our screen results, siRNA targeting actin-related protein 2/3 complex subunit 5 (ARPC5) also inhibited EV71 infection, suggesting a role of the actin cytoskeleton in EV71 infection. The impact of actin polymerization or depolymerization perturbation on virus internalization was further analyzed by treatment with three cell-permeant compounds, latrunculin A, jasplakinolide, and cytochalasin D. All three compounds showed significant interference with EV71(FY)-Luc and EV71 infectivity (Figure 3(g) and (h)). Next, exposing Caco-2 cells to EV71 induced profound reorganization of the actin cytoskeleton, including the formation of dorsal ruffles and microfilaments perpendicular to the surface of the cell, as shown by phalloidin staining (Figure 3(i)), thereby supporting the role of the actin cytoskeleton in EV71 entry and transduction into Caco-2 cells. These data indicate that dynamin 2 and actin cytoskeleton remodelling are involved in EV71 infectious entry in Caco-2 cells.

### EV71 internalization is dependent on the endophilin-a2-mediated endocytic pathway

The above results indicate that EV71 infectious entry into Caco-2 cells is mostly independent of clathrin, caveolin-1, and micropinocytosis but requires dynamin 2 and the actin cytoskeleton. These features are reminiscent of the recently characterized fast endophilin-mediated endocytic (FEME) pathway, which mediates the ligand-triggered uptake of several receptor tyrosine kinases, G-protein-coupled receptor, interleukin-2 receptor, muscarinic acetylcholine receptor 4, and dopaminergic D3 and D4 receptors [24]. Endophilin is concentrated and disassembled on the plasma membrane dynamically when cells are at rest, which is called the priming step of FEME, until stimulation induces the formation of FEME carriers, which is called the activation step [25]. During FEME, triggered by the stimulation of cargo receptors by their cognate ligands, endophilin-positive carriers are rapidly produced, aided by the synergistic actions of actin polymerization and dynamin, and transfer towards the cell centre [24,25].

**Figure 4.**
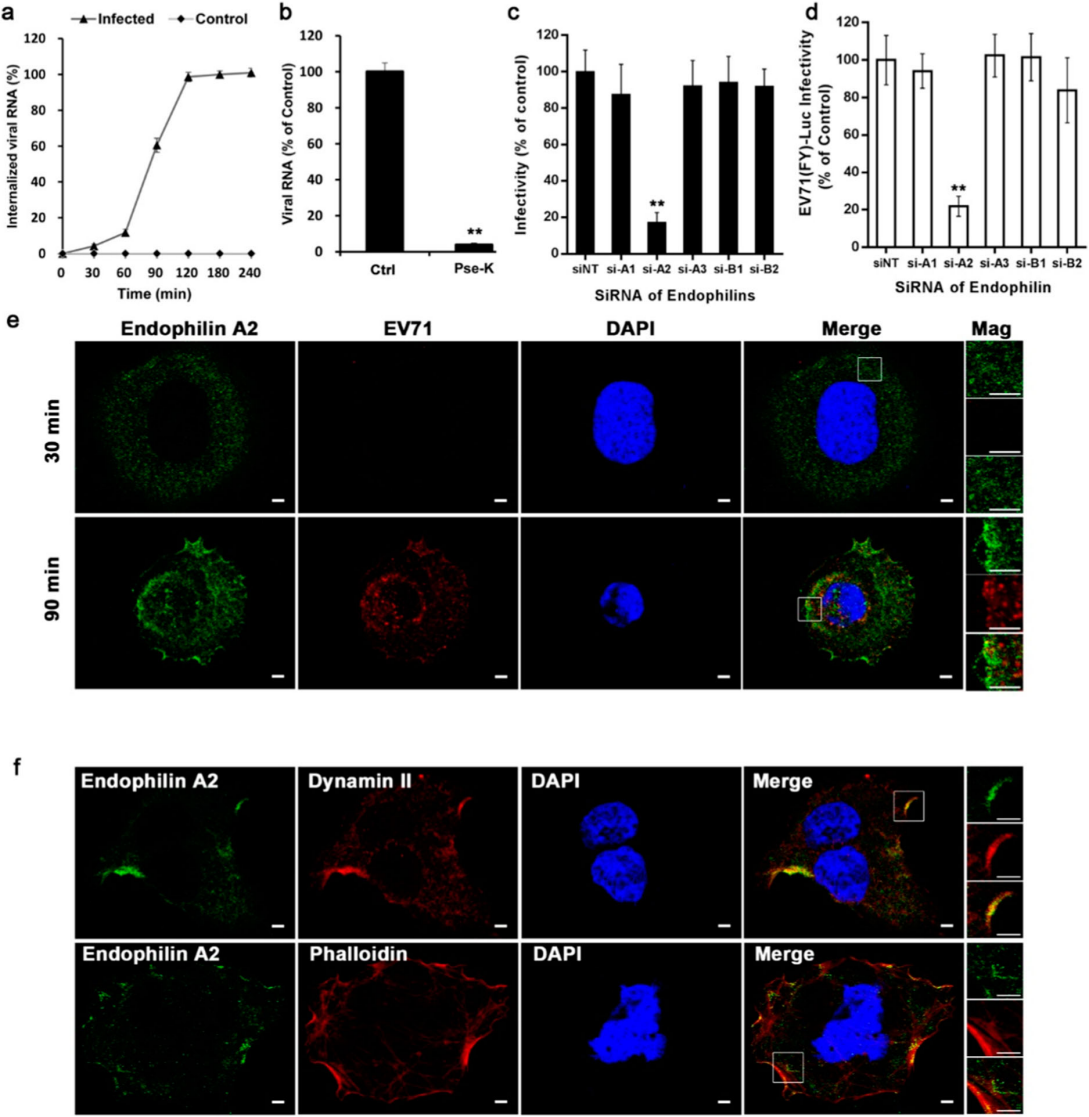
Endophilin-A2-mediated endocytic pathway is critical for EV71 to enter Caco-2 cells (a) Kinetic curve of EV71 entry into Caco-2 cells. Results are shown as a percentage of internalized virus compared with control in which PBS was substituted for proteinase *K*. (b) The effectiveness of proteinase *K* (Pse-K) treatment was evaluated by qRT-PCR. Values are normalized to PBS control (Ctrl). (c, d) EV71 (MOI = 10) and EV71 (FY)-Luc infectivity of Caco-2 cells transfected with siRNA targeting endophilin family members (A1, A2, A3, B1, and B2). Values are normalized to non-targeting siRNA (siNT) control. (e) Confocal microscopy localization of endophilin-A2 (green) with EV71 (MOI = 100) (red) after infected by 30 and 90 min. Nuclei were stained with DAPI. Insets show higher magnification of boxed areas. Scale bars, 20 μm. (f) Confocal microscopy localization of endophilin-A2 (green) with dynamin 2 (red) or actin (red) in Caco-2 cells after EV71 (MOI = 100) infected by 60 min. Insets show higher magnification of boxed areas. Nuclei were stained with DAPI. Scale bars, 20 μm. **p < 0.01.

We first investigated the kinetics of EV71 invasion of Caco-2 cells using a viral entry kinetics assay. As shown in Figure 4(a) and (b), at the beginning of infection (0 min), most EV71 particles could be detached from the cell surface by proteinase *K* (∼96%), revealing the effectiveness of proteinase *K* at detaching virus from the cell membrane. A kinetic curve shows that the internalization of EV71 by Caco-2 cells is stable at early phases (0∼60 min), increases after 60 min, and nearly reaches the maximum after 120 min of incubation. These data indicate that, unlike the rate of FEME, EV71 enters Caco-2 cells in a relatively slow manner.

The most distinctive characteristic of the FEME pathway is the synergistic action of endophilin, the actin cytoskeleton, and dynamin during cargo endocytosis. As the endophilin family comprises five members (A1, A2, A3, B1, and B2), we next explored the roles of these proteins during EV71 entry into Caco-2 cells. Knockdown of endophilin-A2, but not other members, significantly reduced the transduction of EV71(FY)-Luc and the production of EV71 (Figure 4(c) and (d)). At the same time, siRNA targeting endophilin-A2 showed no significant effects on EV71 invading other permissive cells (Fig S4). In addition, subcellular localization analysis of endophilin-A2 and EV71 particles by double staining showed that, 90 min after incubation, decentralized endophilin-A2 accumulated at the ruffles of the cell membrane and the perinuclear area, where endophilin-A2 largely colocalized with EV71 (Figure 4(e)). Moreover, double staining of endophilin-A2 with dynamin 2 or actin revealed that, 60 min after incubation with EV71, endophilin-A2 is colocalized with dynamin 2 at the ruffles of the cell surface and actin at the ruffles and microfilaments (Figure 4(f)). These data suggest an essential role for endophilin-A2, in conjunction with dynamin 2 and the actin cytoskeleton, during EV71 entry into Caco-2 cells. Thus, we conclude that EV71 enters Caco-2 cells mainly and especially via an endophilin-A2-mediated endocytic (EME) pathway, which differs from the recently described FEME pathway in the rate of virus uptake.

Furthermore, as endophilin-A2 has been found to function in membrane scission in clathrin-independent endocytosis [26,27], we then explored whether it acts the same way during EV71 entry in Caco-2. In endophilin-A2-depleted cells, the internalization of EV71 was markedly inhibited as most of EV71 particles remain on the plasma membrane at 90 min post infection (Fig S5). On the plasma membrane (magnified pictures in Fig S5), abundant EV71-containing tubular invaginations (arrowheads) were observed in endophilin-A2-depleted cells compared to negative control siRNA-transfected cells. These findings suggest that endophilin-A2 participate in the membrane scission step of EV71 endocytosis in Caco-2 cells.

### EV71 can be transported through ESCRT-MVBs during infection

**Figure 5.**
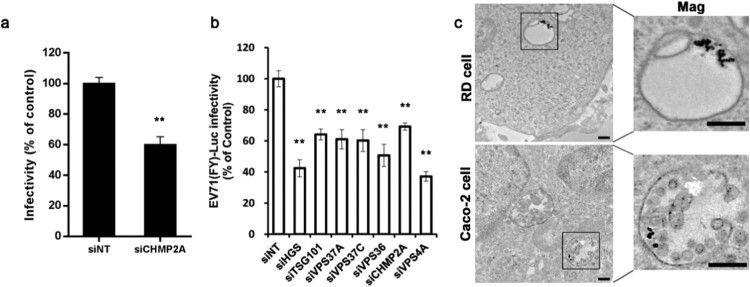
EV71 can be transported through ESCRT-MVBs during infection (a, b) EV71 (MOI = 10) and EV71 (FY)-Luc infectivity of Caco-2 cells transfected with siHGS, siTSG101, siVPS36, siVPS37A, siVPS37C, siCHMP2A and siVPS4A. Values are normalized to non-targeting siRNA (siNT) control. (c) Transmission electron microscopy localization of EV71 particles. RD cells and Caco-2 cells were incubated with EV71 (MOI = 100) for 90 min, fixed, cut into ultrathin cryosections, and stained with EV71/PAG (15 nm). Insets show higher magnification of black boxed areas. Scale bars, 200 ηm. *p < 0.05, **p < 0.01.

Our RNAi screen also revealed that siRNAs targeting HGS/VPS27 (ESCRT-0 subunit), TSG101/VPS23 (ESCRT-I subunit), C13ORF9/VPS36 (ESCRT-II subunit), and VPS4A significantly decreased EV71 infectivity (Figure 1). Hence, we hypothesized that, after endocytosis, EV71 can be transported through ESCRT-MVBs. To test this hypothesis, we utilized siRNAs targeting the ESCRT-III submit (siCHMP2A), which was not in our original screening pool. Knockdown of ESCRT-III also exhibited an inhibitory effect on EV71 infectivity (Figure 5(a)). Pseudotyped virus particles EV71(FY)-Luc were then used to further test the involvement of the components associated with MVB biogenesis in EV71 entry. As shown in Figure 5(b), siRNAs against HGS, TSG101, VPS36, VPS37A, VPS37C, CHMP2A, and VPS4A all decreased viral entry efficiency between 31% and 63%. Therefore, these data demonstrate that ESCRT machinery and VPS4A, both associated with MVB biogenesis, play important roles in EV71 entry into Caco-2 cells. To confirm whether EV71 localizes to MVBs during entry, we infected Caco-2 cells and RD cells with EV71 for 90 min then analyzed virus subcellular localization by transmission electron microscopy (TEM). As shown in Figure 5(c), EV71 particles internalized by RD cells were detected mainly in late endosomes; by contrast, in Caco-2 cells, internalized EV71 particles were detected in MVBs. These data indicate that internalized EV71 particles can be transported through MVBs during the entry stage of infection.

## Discussion

**Figure 6.**
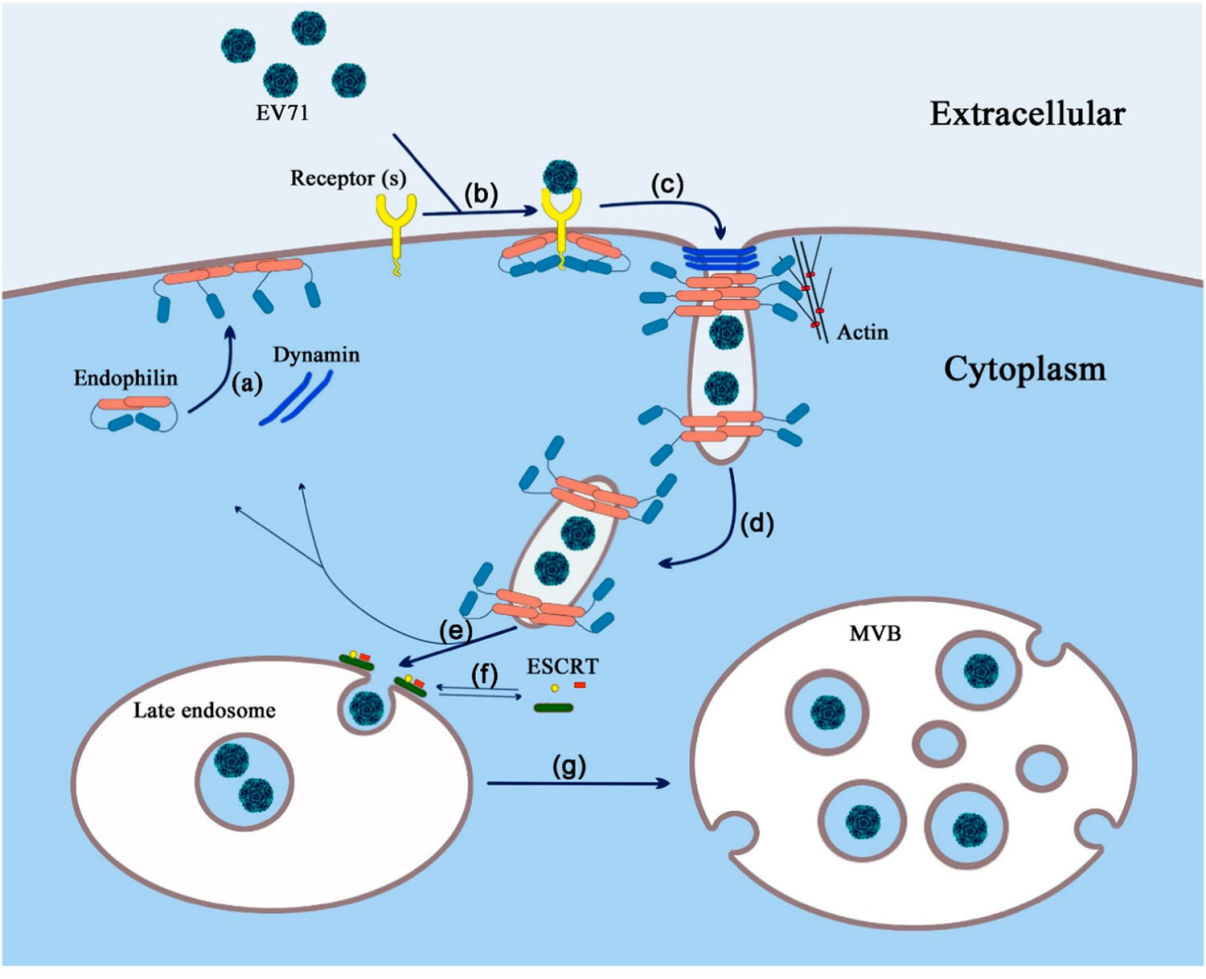
Proposed model of endophilin-mediated EV71 entry route (a) EME priming occurs upon the local pre-enrichment of endophilin on the cell membrane. (b) EV71 attaches to cell surface receptors (such as SCARB2), co-receptors, or other attachment proteins. (c) Endophilin binds to the cytoplasmic motifs of activated receptors with its SH3 domain and mediates the formation of endocytic carriers synergistically with actin polymerization and dynamin. (d, e, g) Endocytic carriers traffic from the cell membrane to the cytoplasm and induce the assembly of ESCRT machinery on late endosomes. The ESCRT complex then mediates the biogenesis of MVBs by catalyzing the invagination of the endosomal limiting membrane. (f) Dynamic assembly and disassembly of ESCRT machinery.

The Caco-2 cell line serves as an in vitro model of the polarized intestinal epithelium and is widely applied in the research of enteroviruses such as coxsackievirus B3 (CVB3), echovirus 7 (E7), EV71, and poliovirus [28–31] E7 was found to enter Caco-2 cells by clathrin-mediated endocytosis and move from early endosomes to late endosomes before viral RNA is released [32]. Although CVB3 and E7 bind to the same receptor on Caco-2 cells, CVB3 enters Caco-2 cells in a dynamin- and clathrin-independent, but caveolin-dependent, mechanism [33]. In this report, we demonstrated for the first time that EV71 enters Caco-2 cells via a clathrin- and caveolin-independent, endophilin-A2-mediated endocytic (EME) pathway and can be transported through ESCRT-MVBs (Figure 6). These findings indicates that enteroviruses are able to take advantage of various cellular internalization mechanisms to infect Caco-2 cells, which offers an explanation as to how these viruses utilize the intestine to initiate infection.

The EME pathway described in this study has the major hallmarks of FEME: it is functionally dependent on the synergistic action of endophilin, actin reorganization, and dynamin but does not require clathrin or caveolin. Interestingly, the endophilin-mediated EV71 entry demonstrated in this study occurs in a relatively slow manner. Generally, in FEME, ligand-triggered uptake of receptors is dependent on a simple ligand–receptor interaction [24]. However, viral attachment is a more complex process and is dependent on the interactions between virions and specific entry receptors, co-receptors, and other entry factors [2,34].

Endophilins are a family of membrane curvature binding proteins that play distinct roles in membrane dynamics and trafficking [35]. In this study, the inhibition of endophilin family members by siRNA shows that endophilin-A2, but not other members, is involved in Caco-2 cells’ internalization of EV71 and EV71(FY)-Luc, especially in the membrane scission process. These results are in agreement with a previous report showing that endophilin-A2 specifically and functionally associates with the membrane scission of clathrin-independent endocytosis [26]. Endophilin is an SH3 (carboxyl-terminal Src Homology 3)-domain-containing protein that can accomplish all phases of building an endocytic vesicle, namely, cargo recruitment, membrane curvature, and membrane scission, which determine its central role in FEME [24]. Its C-SH3 domain binds to the proline-rich motifs (PRMs) present in cytoplasmic parts of activated cargo receptors and mediates adaptor and cargo recruitment [25,36]. Several studies have identified six PRMs in the C-terminal cytoplasmic tail sequence of SCARB2 [37], which enable the SH3 domain of endophilin to recognize SCARB2 and initiate endocytosis. Interestingly, though endophilin-A2 is widely expressed in all tissues, it’s particularly required for EV71 entry in Caco-2 cells, but not other cell types (data not shown). However, we can not rule out the possibility that other endophilin isoforms may be required in other EV71-permissive cells. On the one hand, endophilin family members showed different expression and function in different tissues [38]. On the other hand, EV71 utilizes distinct endocytic pathway in entry different host cells, partly because the diversity of EV71 surface receptor [6]. Further experiments to analyze the attachment mechanism, as well as the exact interaction between endophilin family members and SCARB2 or other putative co-receptors of EV71, are being performed in our laboratory.

It was reported that internalized EV71 particles trafficked from early to late endosomes in RD cells [39]. However, in our siRNA screen, siRNA targeting of Rab7 (marker of late endosomes), but not Rab5 (marker of early endosomes) or Rab11 (marker of recycling endosomes), significantly disturbed EV71 infection, suggesting a minor role for early and recycling endosomes. Further results revealed that MVBs and their sorting machinery (ESCRT) play an important role in EV71 transport in Caco-2 cells. Thus, though we cannot exclude the role of early endosomes in EV71 transport in Caco-2 cells; it is possible that EV71 can bypass early endosomes and directly traffic to ESCRT-MVBs, triggering subsequent events. This hypothesis is consistent with previous research showing that ESCRT-MVBs play a direct role in viral nucleocapsid release to the cytoplasm for replication [13]. This unique transport pattern may result from the interaction between endophilin and ESCRT-associated protein ALIX, a key adaptor of ESCRT-mediated sorting of proteins, which contains the binding site for the conventional SH3 domain of endophilin [40,41]. Furthermore, previous studies proved that SCARB2, also known as lysosome integral membrane protein-2, resides in the membrane of late endosomes and lysosomes, but not early endosomes [42,43], suggesting that the functional receptor may also contribute to the unique transport pattern of EV71. However, the mechanism by which internalized EV71 traffics to ESCRT-MVBs and the role of the MVBs during EV71 uncoating in Caco-2 cells requires additional examination.

In conclusion, our work has uncovered a critical role for clathrin- and caveolin- independent endophilin-A2-mediated endocytosis during EV71 entry into the polarized intestinal epithelial cells line, Caco-2 cells. Our data also suggest that internalized EV71 can be transported through ESCRT-MVBs. To our knowledge, viral entry via EME without the participation of clathrin and caveolin has not been described; therefore, these data may provide an appealing research perspective for other viruses and pathogens. Given that EV71 initiates infection by crossing the intestinal mucosa, the elucidation of its entry into intestinal epithelial cells will provide novel antiviral approaches for the treatment of HFMD.

## Supplementary Material

Supplemental Material
