# Supplementary material for: Endophilin-A2-mediated endocytic pathway is critical for enterovirus 71 entry into caco-2 cells
Source: Emerg Microbes Infect. 2019 May 28;8(1):773–86. doi: 10.1080/22221751.2019.1618686 (PMC6542187; doi:10.1080/22221751.2019.1618686)
Supplement: Supplemental Material [file TEMI_A_1618686_SM1103.docx]

**Supplementary Information**

**Endophilin-A2-Mediated Endocytic Pathway is Critical for Enterovirus 71 Entry into Caco-2 Cells**

Sheng-Lin Chen^1, 2, a^, Yan-Gang Liu^1, a^, Yong-Tao Zhou^1, 3, a^, Ping Zhao^1^, Hao Ren^1^, Man Xiao^2^, Yong-Zhe Zhu^1, *^, Zhong-Tian Qi^1, *^

^1^ Department of Microbiology, Shanghai Key Laboratory of Medical Biodefense, Second Military Medical University, 200433 Shanghai, China

^2^ General Hospital of the Tibet Military Area Command, Tibet 850007, China.

^3^ Company 7, Department of Clinical Medicine, Second Military Medical University, 200433, Shanghai, China

^a^ These authors contribute equally to this work.

^*^Correspondence**:** Zhong-Tian Qi (qizt@smmu.edu.cn) or Yong-Zhe Zhu (zhuyongzhe1984@sina.com), Department of Microbiology, Shanghai Key Laboratory of Medical Biodefense, Second Military Medical University, No. 800, Xiangyin Road, 200433 Shanghai, China, Tel /Fax: +86 218 187 0988

**Key words**: Enterovirus 71, Viral entry, Endophilin, Intestinal epithelial cells, Clathrin, Caveolin

**
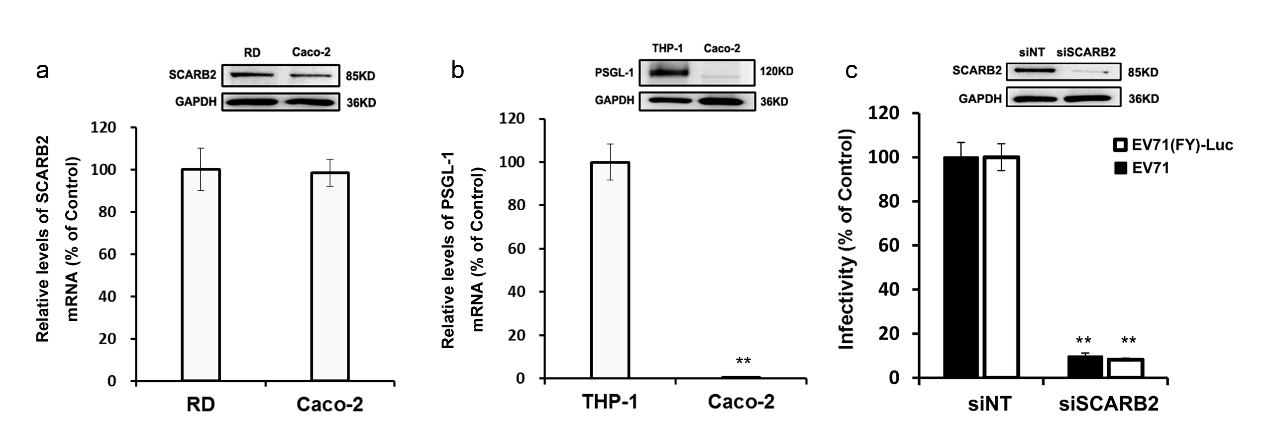
**

**Fig S1. SCARB2 is a functional receptor of EV71 on Caco-2 cells.**

(a, b) Caco-2 cells expressed relatively high levels of SCARB2 but low levels of PSGL1. Real-time PCR and western blot were used to compare the expression level of SCARB2 between RD cells and Caco-2 cells (a) and PSGL-1 between THP-1 cells and Caco-2 cells (b). Values are normalized to control cells (RD or THP-1 cells).

(c) Knock down of SCARB2 showed marked inhibition of EV71 and EV71 (FY)-Luc infectivity. Caco-2 cells were transfected with siRNA of SCARB2 (si SCARB2) then infected with EV71 (MOI=10) and EV71 (FY)-Luc for 48 h. Infectivity was determined by immunofluorescence and firefly luciferase activity, respectively. Values are normalized to non-targeting siRNA (siNT) control. **p < 0.01.

.


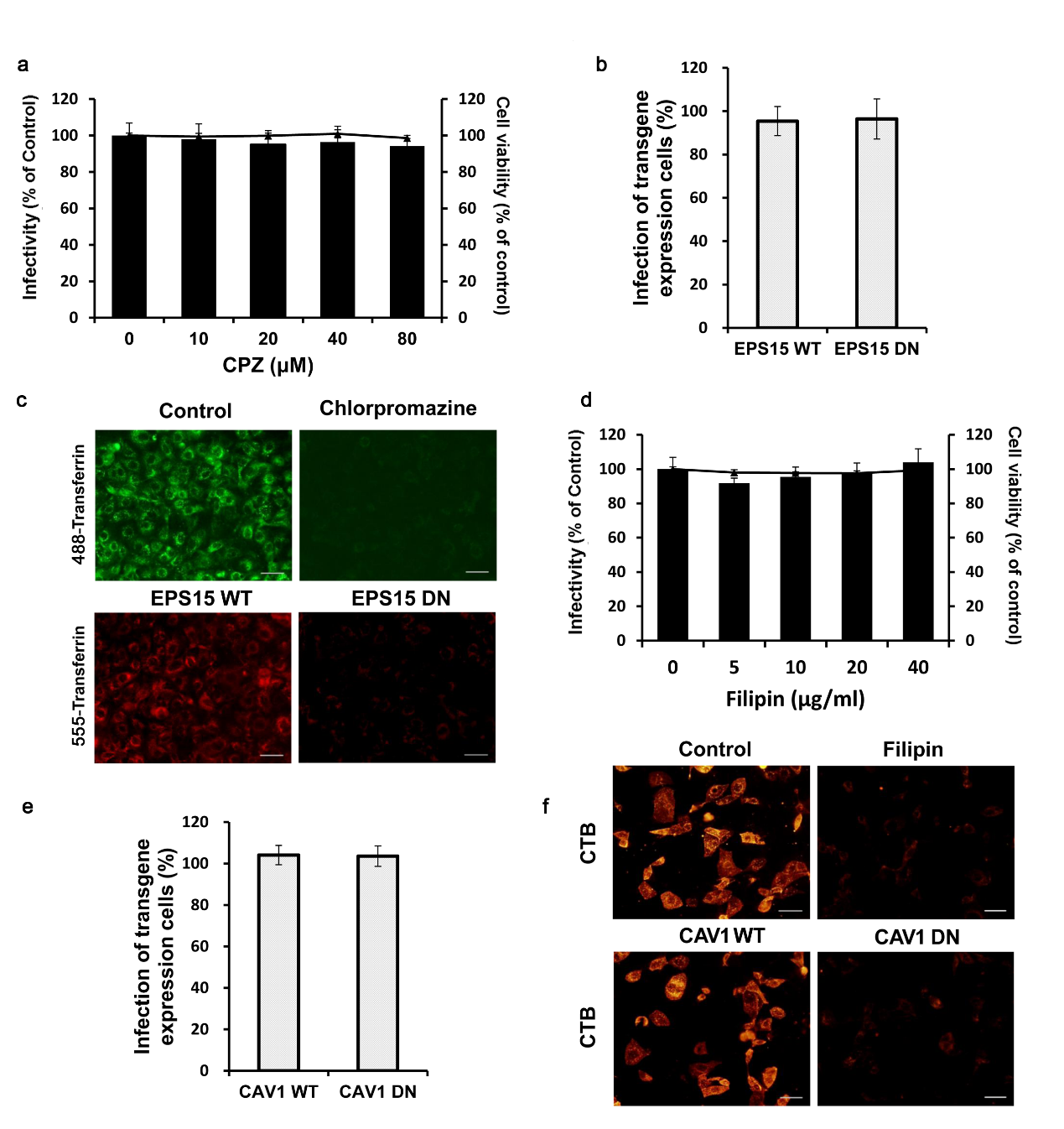


**Fig S2.** **EV71 infection of Caco-2 cells is Clathrin- and caveolin-independent**

(a, b, c) Clathrin-dependent pathway is not required for EV71 infection. Caco-2 cells pre-treated with different concentrations of chlorpromazine (CPZ) (a) or transfected with EPS15 DN (b) were infected with EV71 for 48 h. EV71 infection was assessed by immunofluorescence, and cell viability was tested by CCK-8 assay kit. Values are normalized to DMSO or EPS WT control. As a control, AF-488 transferrin or AF-555 transferrin was added to cells pre-treated with CPZ (80 μM) or cells expressing EPS15 DN and examined by immunofluorescence microscopy. Scale bar, 150 μm (c).

(d, e, f) Caveolin-dependent pathway is not essential for EV71 infection. Caco-2 cells pre-treated with different concentration of Filipin (d) or transfected with CAV1 DN (e) were infected with EV71 for 48 h. Infectivity of EV71 was assessed by immunofluorescence, cell viability was determined by CCK-8 assay kit. Values are normalized to DMSO or CAV1 WT control. As a control, AF-555 cholera enterotoxin subunit B (CTB) was added to cells pre-treated with Filipin (40 μM) or cells expressing CAV1 DN and examined by immunofluorescence microscopy. Scale bar, 150 μm (f).


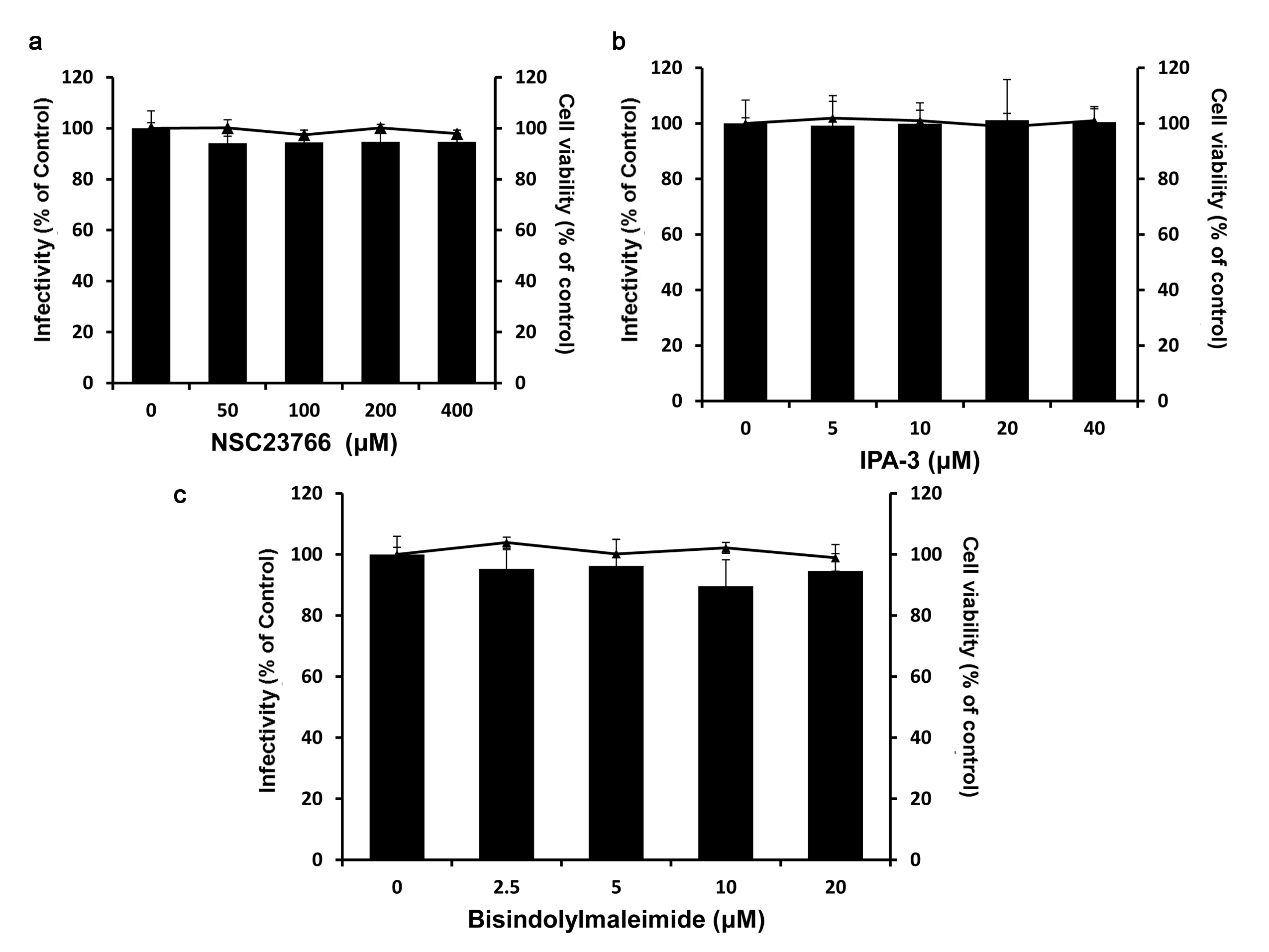


**Fig S3.** **EV71 infection of Caco-2 cells does not involve micropinocytosis**

Caco-2 cells pre-treated with different concentration of NSC23766 (a), IPA-3 (b), and bisindolylmaleimide (c) were infected with EV71 for 48 h. Immunofluorescence was utilized to assess the infectivity of EV71. The CCK-8 assay kit was used to evaluate cell viability. Values are normalized to DMSO control.


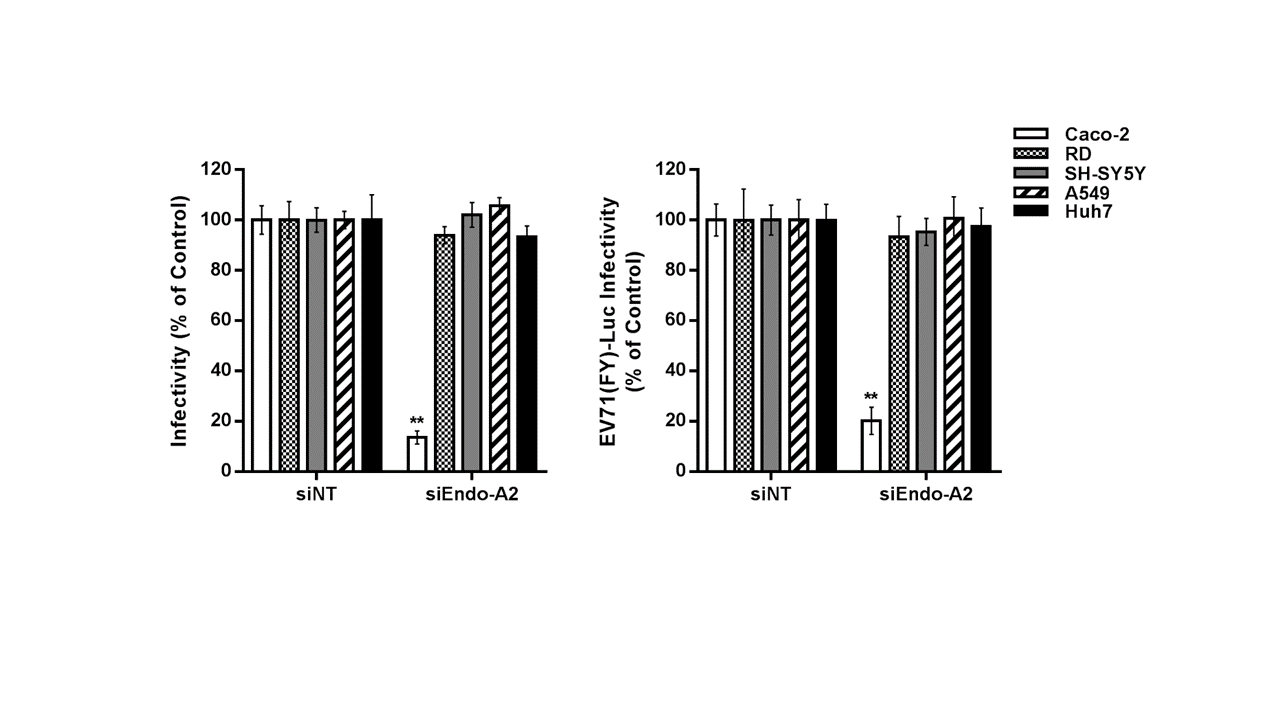


**Fig S4.** **Endophilin-A2 is specifically required for the uptake of EV71 in Caco-2 cells**

Caco-2, RD, SH-SY5Y, A549 and Huh7 cells transfected with siRNA targeting endophilin-A2 (siEndo-A2) were infected with EV71 or EV71 (FY)-Luc for 48 h. EV71 infectivity (left) was measured by immunofluorescence and EV71 (FY)-Luc internalization (right) was analysed by firefly luciferase activity. Values are normalized to non-targeting siRNA (siNT) control. **p < 0.01.


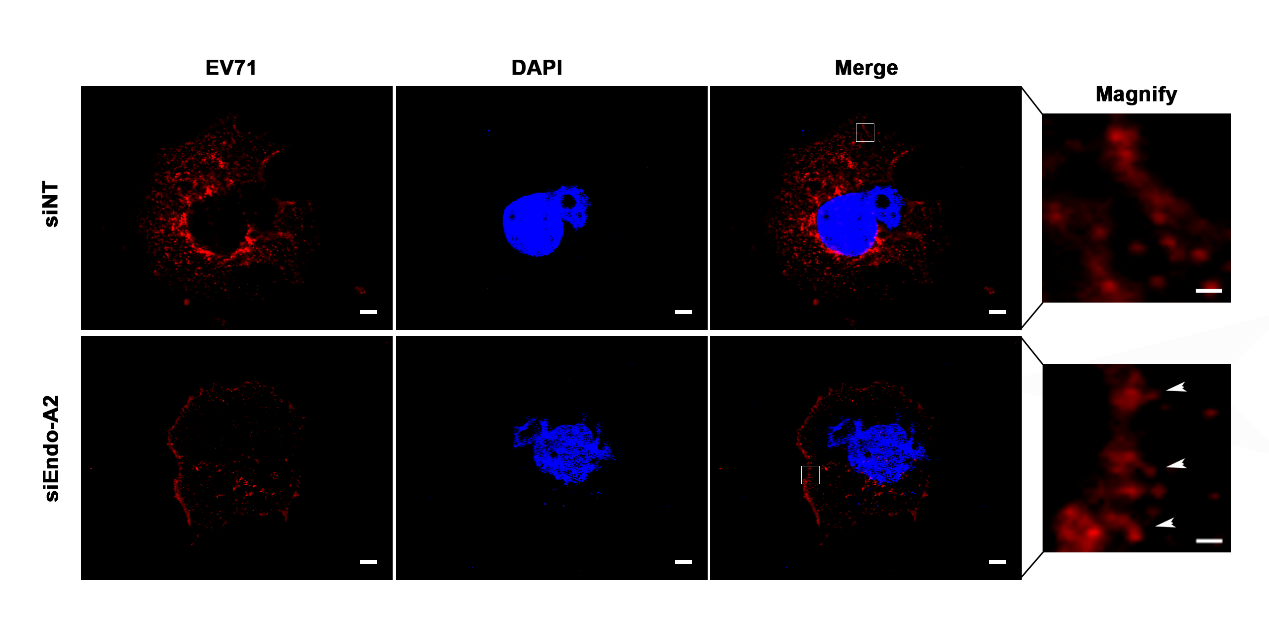


**Fig S5.** **Effect of endophilin-A2 depletion on EV71 endocytosis**

SiEndo-A2 or siNT transfected Caco-2 cells were infected with EV71 for 90 min. After fixation, EV71 particles were labelled with EV71 VP1 antibody and nuclei were stained with DAPI. Insets show higher magnification of boxed areas. Arrowheads indicated the EV71-containing membrane tubular invaginations. Scale bars, 20 μm.

**Table S1. Genes involved in EV71 infection of Caco-2 cells**

| **Genes** | **Gene Accession** | **Name and Function of Genes** | **siRNA Sequence (5’-3’)**  **(D_x_: Duplex Number)** |
| --- | --- | --- | --- |
| AP2B1 | NM_001282 | Adaptor-related protein complex 2, beta 1 subunit serves to link clathrin to receptors in coated vesicles | D_1_: GCAGAUGAGUUACUAGAAA  D_2_: GUAGCGGCAUUAUCUGAAA  D_3_: GCCAAGGGCACAUCUAUAU  D_4_: UCAUGGAAUUCAUCGUAAA |
| ARPC5 | NM_005717 | Actin related protein 2/3 complex, subunit 5 | D_1_: GGAGUAGGGUCCAUUGUUC  D_2_: GAGAGCCCGUCUGACAAUA  D_3_: GUGUGGAUCUCCUAAUGAA  D_4_: GAAUAUGACGAGAACAAGU |
| BECN1 | NM_003766 | Beclin-1 participates in the regulation of autophagy and has an important role in development, tumorigenesis, and neurodegeneration | D_1_: GGAUGACAGUGAACAGUUA  D_2_: UAAGAUGGGUCUGAAAUUU  D_3_: GCCAACAGCUUCACUCUGA  D_4_: UUGAAAACCAGAUGCGUUA |
| C13ORF9 | NM_016075 | Vacuolar protein sorting 36 homolog, a subunit of the endosomal sorting complex required for transport II (ESCRT-II) | D_1_: CAAAGAACAUGGCCAGAUU  D_2_: CAAAGAACCUGGCCCAUUC  D_3_: GGGAAUAGCUAACCCAGUU  D_4_: CGACUGAUUUGGAGAGAUC |
| COPA | NM_004371 | Coatomer protein complex, subunit alpha, one subunit of COP I complex, mediates proteins transport between the endoplasmic reticulum and Golgi compartments | D_1_: ACUCAGAUCUGGUGUAAUA  D_2_: GCAAUAUGCUACACUAUGU  D_3_: GAUCAGACCAUCCGAGUGU  D_4_: GAGUUGAUCCUCAGCAAUU |
| DNM2 | NM_004945 | Dynamin 2, a large GTPase mainly involved in membrane trafficking through its function in the formation and release of nascent vesicles from biological membranes | D_1_: CCGAAUCAAUCGCAUCUUC  D_2_: GACAUGAUCCUGCAGUUCA  D_3_: CCUCCGAGCUGGCGUCUAC  D_4_: AGUCCUACAUCAACACGAA |
| ENTH | NM_014666 | clathrin interactor 1, involved in the formation of clathrin coated vesicles and trafficking between the trans-Golgi network and endosomes | D_1_: GAUCACAGAAUACAGAUAU  D_2_: GAUCAGAGCGUGUUGUUAC  D_3_: UACGAUCCCUGGAAAAUUA  D_4_: GGGAUGAGGAGUGGGAUAA |
| EPN2 | NM_001102664 | Epsin 2, which can interact with clathrin and adaptor-related protein complex 2, alpha 1 subunit | D_1_: AGACUACGCUGUUGGAUUU  D_2_: CCACUGGGCCCUGCAAUGA  D_3_: CCACCAAGCCCGUGUCUGU  D_4_: AUUAAAUCCACUAGAGCGA |
| GAF1 | NM_015470 | RAB11 family interacting protein 5 (class I), which is important for protein trafficking from ARE to the apical plasma membrane | D_1_: GCAACAAGCUGCGCAAGUC  D_2_: GGUACAAGCUGCACUCCAA  D_3_: GUACGUCGGUGGUGGAGAA  D_4_: GGCCAAGAGUAGCUGGUUU |
| GIT1 | NM_014030 | G protein-coupled receptor kinase interacting ArfGAP1 | D_1_: GGACGACGCCAUCUAUUCA  D_2_: GCACACCCAUUGACUAUGC  D_3_: GGACGCCACAUCUCCAUUG  D_4_: CCGCACACCCAUUGACUAU |
| HGS | NM_004712 | Hepatocytegrowth factor-regulated tyrosine kinase substrate, which regulates endosomal sorting and plays a critical role in the recycling and degradation of membrane receptors | D_1_: GCACGUCUUUCCAGAAUUC  D_2_: AGAGAGCGAUGCCAUGUUU  D_3_: GAUAUUCUGUGGAAAGUGU  D_4_: GUAAACGUCCGUAACAAGA |
| NSF | NM_006178 | N-Ethylmaleimide-Sensitive Factor, a key factor for eukaryotic trafficking, including protein and hormone secretion and neurotransmitter release | D_1_: UGGAAAUGCUUAACGCUUU  D_2_: GGUUUAGAAUGCUGCGCUU  D_3_: GUUACAUUAUGAACGGUAU  D_4_: UGGACUAAGUGGAACGUUC |
| PIK3CG | NM_002649 | Phosphatidylinositol-4,5-bisphosphate 3-kinase, catalytic subunit gamma. It is an important modulator of extracellular signals | D_1_: CUACAGCCCUAUCAAAUGA  D_2_: GGUCCAGGCUGUGAAAUUU  D_3_: AGAAAUCUCUGAUGGAUAU  D_4_: GACGUCAGUUCCCAAGUUA |
| RAB3D | NM_004283 | RAB3D, member RAS oncogene family, which can regulate cell secretion | D_1_: GUUCAAACUGCUACUGAUA  D_2_: GUACUGUGGGCAUCGAUUU  D_3_: GGACGAACGUGUUGUGCCU  D_4_: CGGCAGGGACUGUGAUCGA |
| RAB7B | NM_177403 | RAB7B, member RAS oncogene family, which localizes to late endosomes-lysosomes and to the Golgi, regulating the transport between these two intracellular compartments | D_1_: UCACCGACCUGGAGUCUUU  D_2_: UACCAGAGCAUCUUAGAAA  D_3_: UAUCAUAUUGGGUGACACA  D_4_: GAGCCAUUGGUGUGGGAAA |
| RAB7L1 | NM_003929 | RAB7, member RAS oncogene family-like 1, a binding partner of LRRK2, a candidate genes for risk for sporadic Parkinson disease, and part of a complex that promotes clearance of Golgi-derived vesicles through the autophagy-lysosome system. | D_1_: CAGAUUGACCGGUUCAGUA  D_2_: GAGAACGGUUUCACAGGUU  D_3_: GGGACUACAUCAAUCUACA  D_4_: CAACAAGUGUGAUCUGUCC |
| TSG101 | NM_006292 | Tumor susceptibility 101, which plays a role in cell growth and differentiation and act as a negative growth regulator | D_1_: AAACUGAGAUGGCGGAUGA  D_2_: GAACCUCACUGGAACAAUC  D_3_: CCGUUUAGAUCAAGAAGUA  D_4_: UCCCACAGCUCCCUUAUAC |
| VAPB | NM_004738 | VAMP (vesicle-associated membrane protein)-associated protein B, found in plasma and intracellular vesicle membranes, can interact with VAMP1 and VAMP2 and may be involved in vesicle trafficking. | D_1_: GUAAGAGGCUGCAAGGUGA  D_2_: CCACGUAGGUACUGUGUGA  D_3_: UGUUACAGCCUUUCGAUUA  D_4_: GUAAUUAUUGGGAAGAUUG |
| VCP | NM_007126 | Valosin containing protein：The protein encoded by this gene is a member of a family that includes putative ATP-binding proteins involved in vesicle transport and fusion | D_1_: GUAAUCUCUUCGAGGUAUA  D_2_: AAACAGAUCCUAGCCCUUA  D_3_: GAGAGCAACCUUCGUAAAG  D_4_: GCACAGGUGGCAGUGUAUA |
| VPS4A | NM_013245 | Vacuolar protein sorting 4 homolog A ：The protein encoded by this gene is a member of the AAA protein family, associates with the endosomal compartments, and are involved in intracellular protein trafficking | D_1_: GCUGAAGGAUUAUUUACGA  D_2_: UCAAAGAGAACCAGAGUGA  D_3_: GAAUAACAAUGAUGGGACU  D_4_: GAGCCAAGUGCGUGCAGUA |
| SCARB2 | NM_005506 and NM_001204255 | human scavenger receptor class B member 2, which is a receptor of EV71 and serves as a control siRNA in screen assay | D_1_: GAUGAAAUCUUGUCCCUUA  D_2_: UCACUUGACUGGUGGAUAA  D_3_: GGAGAAGACAGUUACCUUA  D_4_: GACAAGUGCAAUAUGAUUA |

**Table S2. List of chemical inhibitors**

| **Inhibitor** | **Specifity** | **concentrations** |
| --- | --- | --- |
| Chlorpromazine | Inhibitor of clathrin-dependent endocytosis | 10 μM, 20 μM, 40 μM, 80 μM |
| 5-ethyl-N-isopropyl amiloride (EIPA) | Inhibitor of Na^+^/H^+^ exchange | 10 μM, 20 μM, 40 μM, 80 μM |
| Dynasore | Cell-permeable, noncompetitive dynamin GTPase activity inhibitor | 10 μM, 20 μM, 40 μM, 80 μM |
| IPA-3 | Inhibitor of PAK-1 activation (irreversible) | 5 μM, 10 μM, 20 μM ,40 μM |
| Filipin III | Disruption of caveolar | 5 μg/ml, 10 μg/ml, 20 μg/ml , 40 μg/ml |
| Bisindolylmaleimide | Inhibitor of protein kinase C | 2.5 μM, 5 μM, 10 μM, 20 μM |
| NSC23766 | RAC1 inhibitor | 50 μM, 100 μM, 200 μM, 400 μM |
| Cytochalasin D | Cell-permeable and potent inhibitor of actin polymerization | 10 μM |
| Jasplakinolide | Promotes actin polymerization and stabilizes actin filaments | 5 μM |
| Latrunculin A | Reversible inhibitor of actin assembly | 5 μM |

**Table S3. Sequences of primer pairs specific to target gene**

| **Target Gene** | **Sequences of qRT-PCR Primer Pair (5’-3’)** |
| --- | --- |
| **GAPDH** | Forward Primer TGGGCTACACTGAGCACCAG  Reverse Primer AAGTGGTCGTTGAGGGCAAT |
| **SCARB2** | Forward Primer GGCCGATGCTGCTTCTACA  Reverse Primer GGTCTCCCCTCTGAGGATCTC |
| **PSGL-1** | Forward Primer CTACCAAAAGAGGTCTGTTCATAC  Reverse Primer TGTGCAGGGTGAGGTCATC |
